# Advances and Future Challenges in Recombinant Adenoviral Vectored H5N1 Influenza Vaccines

**DOI:** 10.3390/v4112711

**Published:** 2012-11-01

**Authors:** Jianfeng Zhang

**Affiliations:** Vaxin Inc, 19 Firstfield Road, Suite 200, Gaithersburg, Maryland 20878, USA; Email: zhang@vaxin.com; Tel.: +1-240-654-1450 (ext. 12); Fax: +1-855-557-1369.

**Keywords:** recombinant adenoviral vector, influenza vaccine, cross-protection, pandemic influenza, H5N1

## Abstract

The emergence of a highly pathogenic avian influenza virus H5N1 has increased the potential for a new pandemic to occur. This event highlights the necessity for developing a new generation of influenza vaccines to counteract influenza disease. These vaccines must be manufactured for mass immunization of humans in a timely manner. Poultry should be included in this policy, since persistent infected flocks are the major source of avian influenza for human infections. Recombinant adenoviral vectored H5N1 vaccines are an attractive alternative to the currently licensed influenza vaccines. This class of vaccines induces a broadly protective immunity against antigenically distinct H5N1, can be manufactured rapidly, and may allow mass immunization of human and poultry. Recombinant adenoviral vectors derived from both human and non-human adenoviruses are currently being investigated and appear promising both in nonclinical and clinical studies. This review will highlight the current status of various adenoviral vectored H5N1 vaccines and will outline novel approaches for the future.

## 1. Introduction

Influenza is a contagious acute respiratory disease that remains a serious public-health problem today [1,2] and results in substantial economic burden every year [3], even though most influenza virus infections are self-limited. There are three type influenza viruses (A, B, and C). All of them can infect humans, but influenza A viruses are the most virulent types of responsible for pandemics [4]. Influenza A viruses can be divided into subtypes based on antigenic differences in their surface glycoprotein hemagglutinin (HA) and neuraminidase (NA). Currently, 17 HA and 9 NA subtypes of influenza A viruses have been described [5,6]. The genome of influenza A viruses comprise eight single–stranded, negative-sense RNA segments that encode eleven proteins. These viruses are continuously evolving through accumulated mutations (antigenic drift) and genetic reassortment (antigenic shift) that results in the emergence of new strains. An influenza pandemic may take place when a new strain of influenza A virus carrying novel HA and/or NA genes enters the general population with little or no immunity against it.

Compelling evidence suggests that avian influenza viruses (AIV) have contributed with genetic material to pandemic influenza strains that hit the world in 1918 (‘Spanish flu’), 1957 (‘Asian flu’), 1968 (‘Hong Kong flu’), and 2009 (‘Mexican flu’) [7,8,9]. Currently circulating highly pathogenic avian influenza (HPAI) H5N1 virus poses another potential pandemic threat to humans [10]. This virus emerged in 1996 and the first confirmed direct transmission of AIV to humans without an intermediate host was in 1997 during a poultry outbreak in Hong Kong [11,12]. After re-emergence in 2003, the H5N1 virus strains have spread from Asia to Europe and Africa and has caused severe disease in poultry and wild birds in multiple countries [13]. These viruses crossed the species barriers infecting mammals, including domestic cats, Owston’s civets, leopards, tigers, dogs, stone martens, pigs, plateau pika, and humans [13]. As of August 2012, there have been a total of 608 confirmed human cases of H5N1 infection, with 359 deaths (59% mortality rate) in 15 countries since 2003, as reported the World Health Organization [14]. Although up to now H5N1 viruses have not yet shown efficient transmissibility among humans, the transmissions of H5N1 viruses from human to human have been documented in several countries [15]. Two recent laboratory-engineered mammalian-transmissible H5N1 virus strains [16,17] and additional surveillance data [18] clearly indicate that HPAI H5N1 virus can evolve into a strain capable of human-to-human transmission, which may induce a global disaster because the human population, overall, has no immunity against this virus. In addition, a human–to–human transmissible HPAI H5N1 virus has the potential of being utilized as a bioterrorist weapon [19].

## 2. The Need for Better H5N1 Pandemic Vaccines

To mitigate the spread of this contagious virus and reduce the degree of pathogenicity in infected hosts, there is a critical need for an effective H5N1 pandemic preparedness plan [20], that likely requires a combination of pharmaceutical prophylaxis, treatment (vaccines and antiviral drugs) and non-pharmaceutical interventions (general personal hygiene and reduction of nonessential contacts) [21,22]. H5N1 pandemic vaccine development is considered to be the cornerstone of pandemic influenza control and prevention.

Traditional inactivated or live attenuated vaccines are somewhat effective in protecting people against seasonal influenza by targeting HA. However, it is difficult to produce sufficient amounts of effective H5N1 pandemic vaccines in a timely manner using the conventional egg-based system because: (i) It takes at least four months to produce the first vaccine after the identification of a new potential strain [23]; (ii) H5N1 viruses are highly lethal to personnel [14], requiring biosafety level 3 containment facilities for vaccine production; (iii) H5N1 viruses do not replicate well in chicken embryos, resulting in low yields of the H5N1 vaccine virus per egg [24]. In addition, the supply of eggs for vaccine production might be compromised during an H5N1 pandemic when many chickens are infected or culled. In general, both inactivated and live attenuated H5N1 vaccines are only mildly immunogenic in humans, requiring high doses of antigen, multiple cycles of vaccination, and/or the use of adjuvants [13,25,26]. Furthermore, the live attenuated influenza vaccine (LAIV) is only licensed for healthy people from 2 through 49 years of age and excludes high-risk groups [27]. Overall, the platforms that are licensed for the existing seasonal influenza vaccines are not optimal for an H5N1 pandemic scenario as experienced in the 2009 H1N1 influenza pandemic [28]. Thus, it is urgent to explore alternative pandemic influenza vaccine strategies capable of preventing and controlling H5N1 infection in a timely manner.

Several egg-independent vaccine strategies, such as mammalian cell-based vaccines, recombinant protein-based vaccines, virus-like particle (VLP)-based vaccines, DNA vaccines, bacterial vectored vaccines, and viral vectored vaccines, have been extensively studied as alternative approaches [28,29,30]. Included in the list of alternative strategies are the recombinant adenoviral (rAd) vectored H5N1 vaccines, which are promising candidates that induce rapid and long-term cross-protective immunity against continuously evolving H5N1 viruses [31,32,33,34,35].

## 3. Recombinant Adenoviral Vectors for Vaccination

Since the first isolation of adenovirus (Ad) from human adenoid tissue culture nearly 60 years ago, numerous adenoviruses (Ads) have been isolated from a variety of animal species and humans [36,37]. Based on serological properties and genome DNA sequences, human Ads have been classified into 53 distinct serotypes and grouped into 7 subgroups (A–G) [38], with the majority causing only mild clinical symptoms such as colds or gastroenteritis [39]. Ads are non-enveloped icosahedral DNA viruses of about 70 to 100 nm in diameter [40]. Ad viral genome, a linear double-stranded DNA of about 33 to 38 kb, is flanked by two inverted terminal repeats (ITRs) and its packaging signal sequence (ψ) locates adjacent the left ITR [41]. This genome is tightly associated with viral core proteins and packaged in a naked icosahedral capsid which composes three major structural proteins, hexon, penton, and a knobbed fiber, along with a number of other minor proteins [42]. Based on the transcription, which occur either before or after replication of viral DNA, the genome can be divided into early (E1a, E1b, E2a, E2b, E3, and E4) or late (L1-L5) regions [41,43].

In 1977, Graham and colleagues developed a system for the production of replication defective rAd vectors in a helper free environment. This system was based on the human embryonic kidney (HEK) 293 packaging cell line, which provided E1 gene products in trans [44]. Since then, rAd vectors have been extensively investigated for their applications in vaccine development. There are numerous noteworthy reasons for utilizing a rAd as a vaccine delivery vector: (i) the safety of rAd based vaccines has been demonstrated in nonclinical and clinical studies against a number of infectious diseases; (ii) the techniques are well established for rAd vaccine construction and large-scale production in well characterized suspension cells (*i.e.* PER.C6) in a very cost effective manner [32,45]; (iii) novel formulations have allowed rAd vectored vaccines to be stored in liquid buffer [46] or as lyophilized dry power [47] at 4 ^o^C for at least one year and newly developed thermostabilization techniques may allow the storage of rAd vectors at room temperature at up to 45 ^o^C for 6 months with minimal declining infectivity [48]; (iv) unlike unnatural adjuvants (manmade molecules and/or administration to an inappropriate site) that may induce unpredictable consequences [49], rAd vectored vaccines can provide ‘self-adjuvanting’ activity [50] by activating innate immunity [51], which may lower regulatory and commercialization hurdles; and (v) rAd vectored vaccines can be administered by various routes [52,53,54,55,56] because they can infect a wide variety of cell types and tissues in both dividing and non-dividing cells, including antigen-presenting cells [31].

Both replication-competent and replication-defective rAd vectors have been used as vaccine carriers in early studies [57,58,59,60]. Due to the fact that human Ad serotype 5 (Ad5) is the best characterized of all Ads in virus-cell interaction, DNA replication and transcription, protein expression, and viral assembly [41], it is thus not surprising that the majority of currently used rAd vectors are derived from Ad5.

Three generations of rAd vectors based on Ad5 have been created and tested. The first-generation rAd vectors are E1 or E1/E3 deleted vectors which are generated and propagated in packaging cells, such as HEK 293 and PER.C6 cells, that supply the products of the deleted genes in trans. The disadvantage with the HEK 293 cells is that replication-competent adenoviruses (RCA) can be generated within rAd vector productions through homologous recombination between identical sequences framing the E1 locus displayed by HEK 293 cells and the rAd vector backbone [61]. The presence of RCA in rAd vaccine stocks raises the possibilities of undesired Ad infection, amplification, and mobilization. It also triggers the host immune response and gives rise to inflammation and tissue damage [62,63]. RCA-free rAd vectors can be constructed and produced by using the PER.C6 cell line and its matched plasmids [32,64]. PER.C6 is one of the most comprehensively documented mammalian cell lines, with an excellent safety record and a current biologics master file on record with the FDA [65]. The standard operating procedures for large-scale production of RCA-free first-generation rAd vectors by using PER.C6 cells for clinical trials are well established [32,66,67].

In an attempt to reduce vector toxicity, increase their cloning capacity, and prolong transgene expression *in vivo*, several investigators designed second-generation rAd vectors by further deletion of some or all of E2 and/or E4 genes in the E1 or E1/E3 deleted vector backbone [68,69,70,71]. The third-generation rAd, which are helper-dependent adenoviral vectors (HDAd), lacks all or nearly all viral coding sequences except necessary cis-acting elements: the packaging signal sequences (ψ) and two ITRs [72]. Although the second or third-generation rAd vectors might have the advantage of evading any pre-existing anti-vector immunity and inducing better immune responses [73,74], they remain difficult to produce and purify for clinical application.

One potential problem with rAd vectors derived from Ad5 is that the majority human populations have pre-existing immunity to Ad5 as a result of natural exposure [75,76] that can severely reduce the potency of injected Ad5 vectored vaccines [77,78,79]. Several strategies to circumvent this potential drawback have been studied including the development of rare serotype human rAd vectors, non-human rAd vectors, and molecular engineered Ad5 vectors.

A number of novel rAd vectors based on the rare serotype human Ads (*i.e.* Ad11, Ad26, Ad35, Ad48, Ad49, and Ad50) [80,81,82,83,84] and non-human rAd vectors such as chimpanzee Ads [85,86,87], bovine Ad 3 [88], canine Ad 2 [89], and porcine Ad 3 [90] have been examined as alternative vectors for evading pre-existing Ad5 immunity. However, studies have clearly showed the transgene product-specific antibody responses induced by the vectors derived from both the rare human serotype viruses and chimpanzees viruses are markedly lower than those induced by Ad5 based vectors [83,91,92]. Moreover, the pre-existing Ad5-specific CD4+ T and CD8+ T cells may still have a negative impact on the potency of a chimpanzee rAd vectored vaccine [93,94]. Given the impressive immune responses generated by Ad5 based vectors in naïve animals, several investigators have constructed molecular engineered Ad5 vectors to minimize the effect of the anti-Ad5 immunity [95,96]. Despite the substantial progress in molecular engineering of rAd vectors that has been made since the initial studies, these vectors have been proven difficult to construct and produce. In addition, the safety of the rare serotype human rAd vectors, non-human rAd vectors, and molecular engineered rAd vectors needs to be investigated before their use in humans [97].

## 4. Adenoviral Vectored Nasal Vaccines Can Bypass Pre-Existing Immunity

Immunization with rAd vectored vaccines by different routes and doses can have a significant effect on the type and strength of the induced immune responses [98,99,100,101]. Similar to traditional vaccines, rAd vectored vaccines are usually delivered parenterally to stimulate humoral and cellular immune responses. However, the efficacy of the parenterally delivered rAd vectored vaccines may have interference by the presence of pre-existing Ad5 immunity. In contrast, administration of rAd vectored vaccines via alternative routes might overcome pre-existing immunity against the Ad5 vector [53,55,102,103,104,105].

There are evidences that intranasal immunizations with rAd vectored vaccines can overcome the effect of pre-existing immunity to Ad5 vectors. They also induce sufficient immune response against encoded antigens and provide protection against challenge of pathogens in mice, rabbits, and non-human primates [106,107,108,109,110,111]. These events could be caused due to high-efficiency gene delivery into cells in the superficial layer along the mucosal barrier, together with the potent antigen presentation associated with this immunocompetent interface tissue.

Although intranasal inoculation of rAd vectors in mice or rats could lead to some viral dissemination to the olfactory bulb and the central nervous system [112,113,114,115], no cytopathic effect was detected in the central nervous system structures [112]. After intranasal delivery of a rAd vectored anthrax vaccine in rabbits, the rAd vector DNA was transiently detected in trachea, olfactory bulb, and lymph node. However, no Ad vector DNA was found in liver, blood, and brain, but the majority of DNA was detected in the lung and could persist there for up to a month (Vaxin unpublished data). Intranasal administration of a rAd vectored vaccine had no undesirable systemic effects in rabbits, cynomolgus macaques or humans [53] (Vaxin unpublished data).

The natural tropism of Ad5 vectors for the respiratory tract makes them useful for the purpose of intranasal vaccination against pathogens (*e.g*. influenza virus), that preferentially initiate infection at the mucosal site. Thus, intranasal immunization of Ad vectored vaccines through the use of a nasal spray could be advantageous since immunization becomes simple, practical, economical, and well suited for mass vaccination campaigns.

## 5. Adenoviral Vectored H5N1 Vaccines

### 5.1. Multifaceted Immune Responses

Comparative data have demonstrated that rAd vectored vaccines induced better humoral and cellular immune responses than recombinant protein vaccines, plasmid-based DNA vaccines, and other recombinant vector systems currently available [83,116,117]. The effectiveness of rAd vectored influenza vaccines has been intensively evaluated in a series of efforts focused on the development of vaccines against various influenza virus subtypes, particularly in HPAI H5N1 vaccines. 

In 2006, Gao *et al.* reported that a rAd vector encoding HA gene from A/Vietnam/1194/04 elicited robust humoral and/or cellular immune responses in vaccinated mice and chickens. Immunized animals were completely protected from a lethal challenges with H5N1 (A/Vietnam/1194/04) [118]. They also described that mice immunized with a rAd vector containing HA2 domain from A/Vietnam/1194/04 generated negligible humoral neutralizing response but strong cellular immunity. Also, animals were partially protected when challenged with a lethal dose of A/Vietnam/1194/04. In addition, vaccination with a rAd vector encoding HA1 domain from A/Hong Kong/156/97 could provide cross-protection against the antigenically distinct A/Vietnam/1194/04 virus [118]. At the same time, Hoelscher *et al.* observed that mice intramuscularly or intranasally immunized with a rAd expressing HA protein from A/Hong Kong/156/97 were effectively protected against lethal challenges with the heterologous (A/Hong Kong/483/97) and (A/Vietnam/1203/04) H5N1 influenza viruses, even without a strong humoral neutralizing response against A/Vietnam/1203/04 virus [119]. These two studies highlight that robust cellular immune responses induced by rAd based influenza vaccines have the advantage of conferring broader protection against continuously evolving H5N1 viruses. Cellular immune response also plays an important role in virus clearance and promoting early recovery during H5N1 infection [120,121]. 

In addition, a rAd vaccine encoding a HA gene is able to stimulate cross-protective immunity between different subtypes of avian influenza virus [52,122,123]. This suggests that rAd based vaccination may induce secretion of antibodies against conserved epitopes of HA molecules from different subtypes or strains [124], providing cross-protection against divergent influenza viruses.

During the last ten years, the research group at Vaxin has been developing RCA-free, rAd based nasal influenza vaccines [32,53,125]. Studies from Vaxin and others have demonstrated that a single-dose intranasal administration of a rAd vectored influenza vaccine could confer protection against virulent influenza virus challenges in animals [126,127] (Vaxin unpublished data). Growing evidence suggests that rAd vectored nasal influenza vaccines induce greater antigen-specific IgA and IgG responses in the respiratory tract. These vaccines could also provide more virus-specific activated T-cells in the lung and better protection than intramuscularly injected rAd vaccines [123,128,129]. This is significant because mucosal immunity can potentially provide cross-protection against different strains of influenza [130,131,132,133,134,135]. The more relevant immune responses are found in human clinical trials where human subjects could be safely and effectively immunized with rAd vectored nasal influenza vaccines even in the presence of pre-existing anti-Ad5 immunity [53] (data of a recent rAd H5N1 nasal vaccine phase I unpublished clinical study involving 48 human).

Recently and unexpectedly, Vaxin found that significant protection is afforded by a single intranasal vaccination but not intramuscular injection of either the AdNCHA1.1 vaccine (a rAd vector encoding codon-optimized HA1 domain from A/New Caledonin/20/99) or an AdE vector (a rAd vector without the transgene) against A/California/04/09 (pandemic H1N1) or A/Puerto Rico/8/34 viruses after two days administration [136]. Although the mechanism is not yet clear, this observation suggests that intranasal immunization of a rAd vector expressing HA proteins may confer seamless protection against influenza virus infection by eliciting of innate as well as adaptive immune responses. More recently, we found that the intranasal administration of an AdVNHA5 vector (a rAd vector encoding codon-optimized HA gene from A/Vietnam/1203/04) could provide similar protection against A/vitenam/1203/04 virus challenge infection in mice (Vaxin unpublished data).

Similar to other rAd based vaccines [137,138,139,140], the rAd vectored H5N1 vaccines are able to induce rapid and long-lasting humoral and cellular protective immunity in mice [127,129,141]. This is a highly desirable attribute for an H5N1 vaccine to overcome the emergence of an H5N1 pandemic. The establishment of a complete protection could limit future infection and reduce transmission rates by providing a long lasting immunity to the herd.

### 5.2.Strategies for Broad Protection

The HA glycoprotein is the primary influenza vaccine target that stimulates higher levels of HA inhibition (HI) and neutralizing antibodies (NAB) titers, stronger cellular immune responses, and confers better protection against homologous or heterologous H5N1 virus challenge than NA, nucleoprotein (NP), matrix protein 1 (M1) or M2 based vaccines [142,143,144,145]. However, a monovalent HA rAd based vaccine may not provide adequate protection against a broad range of heterologous strains of H5N1 influenza viruses, that are currently classified into more than ten antigenically unique clades on the basis of phylogenetic analysis of their HA genes [146]. 

Three approaches, which are not mutually exclusive, have been tested in animals to evaluate the efficacy of rAd based H5N1 vaccines against H5N1 isolates from various clades: (i) co-immunization with multivalent rAd vectors expressing HA glycoproteins or other antigens derived from different clades; (ii) a rAd vector expressing HA protein with NA protein and/or other highly conserved influenza antigens; (iii) different combination of prime-boost with rAd based H5N1 vaccines.

The efficacy of a multivalent rAd based HA vaccine was first examined by immunizing mice with rAd vectors encoding HA genes from clade 1 virus (A/Vietnam/1203/04), or rAd vectors encoding HA genes from clade 2 virus (A/Indonesia/5/05), or both [147]. Serum tests showed that mice receiving clade 1 vaccine or clade 2 vaccine produced strong neutralizing antibodies against H5N1 viruses of the same clade but not against the other clade. The mice that received both vaccines generated protective immunity against H5N1 viruses from both clades, but antibody titers were relatively low [147]. In a similar study, mice immunized with both rAd vectors containing A/Hong Kong/482/97 (clade 0) HA and rAd vectors encoding A/Vietnam/1194/04 (clade 1) HA produced similar HI antibody titers against A/Vietnam/1194/04 to the mice immunized with clade 1 vaccine alone, but generated significantly higher HI titers than the mice only received clade 0 vaccine [148]. These results demonstrated that multivalent rAd based H5N1 vaccines could be mixed and delivered simultaneously. The immune response elicited by different rAd vectors suggests that some H5N1 strains may provide better protection against heterologous challenge than others. Interestingly, Hoelscher *et al.* showed that co-administration a rAd vector expressing HA gene with a rAd vector expressing NP gene could further enhance the efficacy of the vaccine [147]. By contrast, Petal *et al.* found that the immunization a rAd based HA vaccine together with a rAd vector expressing NP actually damaged the quality of the protective immune responses [149]. Future studies are needed to focus on what are the relative benefits or detriments of combinations of multivalent rAd vaccination.

Several groups have tested the feasibility of a rAd H5N1 vaccine encoding multiple influenza genes to elicit heterosubtypic immunity. Holman *et al.* constructed two rAd vectors expressing HA, NA, and M1 genes from a clade 1 H5N1 virus A/chicken/Thailand/CH-2/04 (cAdVax-FluAv) and from the 1918 Spanish influenza strain A/South Carolina/1/18 (cAdVax-Flusp) respectively [150]. These vaccines are based on a second generation rAd platform that had been applied to several disease models [102,151,152]. The cAdVax-FluAv vaccine induced HI titers against both clade 1 (A/Vietnam/1203/04) and clade 2 (A/Indonesia/5/05) viruses, with a higher results of immune response against the more homologous clade 1 virus. The same vector induced a 100% protection in mice challenged with both clade 1 and clade 2 viruses [150]. Although the rAdVax-Flusp vaccine did not elicit HI antibodies against A/Vietnam/1203/04 and A/Indonesia/5/05 viruses, immunization with rAdVax-Flusp did provide complete protection against a lethal A/Vietnam/1203/04 virus challenge and partial protection against A/Indonesia/5/05 challenge [150]. Park *et al.* created a rAd H5N1 vaccine (rAdv-AI) expressing two fusion genes linked by an internal ribosome entry site (IRES) based on a traditional first-generation rAd vector [128]. One fusion gene contains three copies of M2e (2-24 amino acids of M2), the extracellular domain regions of the HA gene of A/Hong Kong/213/2003, and human CD40L. Another fusion gene carries M1 and M2 genes from A/Vietnam/1203/2004. In contrast to intramuscular immunization only eliciting systemic immunity, intranasal vaccination with the rAdv-AI vaccine was able to induce both systemic and mucosal antigen-specific immune responses [128], that may be more effective against antigenically distinct H5N1 viruses. Interestingly, mice received the rAdv-AI vaccine were completely protected against a lethal H5N2 virus challenge. However, -no anti-H5N2 neutralizing antibodies were detected [128]. More recently, Pandey *et al.* reported that a rAd vector encoding both HA and NP genes from A/Vietnam/1203/04 could induce protective immune response in mice, even with high levels of pre-existing Ad5 immunity [105]. The use of a rAd H5N1 vaccine encoding multiple influenza antigens may have several advantages, like: the reduction of production costs, the decrease of vaccination side effects and the induction of balanced immune responses to all antigens. 

In a series of studies, Epstein and her colleges demonstrated that a DNA priming-rAd boosting regimen focusing immunity on conserved influenza virus antigens (NP and/or M2) is able to stimulate a broadly protective immunity against challenge with virulent heterologous viruses, including different H5N1 strains, in both mouse and ferret models [129,153,154,155]. They further showed that intranasal rAd boosting could induce stronger mucosal immunity, enhance viral clearance, and confer better protection against morbidity following highly virulent H1N1 and H5N1 virus challenge. These results were superior compared to intramuscular rAd boosting or intranasal cold-adapted influenza virus boosting [129]. However, vaccines based on conserved antigens are not capable of replacing HA antigen matched vaccines because these vaccines alone cannot prevent viral infection. Therefore, only reduce the severity of disease and protect animals against low dose influenza virus challenge [156]. A recent study suggested that NP and M2 antigens may require combinatorial vaccination with HA antigens to become suitable candidates for universal influenza vaccines [142]. Using HA molecules from different subtypes of the influenza viruses, Wei *et al.* demonstrated that DNA priming-rAd boosting vaccination could generate a strong antigen-specific cellular immunity and high titers of broadly neutralizing antibodies, including antibodies against conserved HA2. They also protect animals against challenge with more divergent influenza strains [52], even in animals with pre-existing influenza immunity [157]. rAd vectors have been demonstrated to be very immunogenic at priming [117] and boosting [158]. In agreement with these studies, Lin *et al.* recently showed that a rAd encoding HA gene priming followed by a recombinant trimetric HA protein boosting elicited a robust neutralizing antibody responses against homologous and heterologous H5N1 virus strains [159]. The prime-boost immunization strategies are excellent regimes for enhancing the potency of rAd based H5N1 vaccines, but they require multiple doses over a prolonged immunization schedule, which might limit their usefulness during an H5N1 pandemic.

### 5.3. Human Adenovirus Serotype 4 Vectored H5N1 Vaccines

In addition to Ad5 derived vectors, replicating recombinant adenovirus serotype 4 (rAd4) vector has also been studied for prevention of several disease models [160,161]. Recently, Alexander *et al.* presented nonclinical data that intranasal administration of a rAd4 encoding HA gene from A/Vietnam/1194/2004 induced HA specific HI antibodies and cellular immune responses. This adenoviral construction also protected mice against a lethal H5N1 reassortant viral challenge even in the presence of pre-existing anti-Ad4 immunity [162]. One phase I clinical trial designed to evaluate the safety and immunogenicity of an oral rAd4 H5N1 vaccine has been completed [163]. Another phase I trial in healthy adults aged 19-45 years to determine the optimal route and dose for the rAd4 H5N1 vaccine is underway [163].

### 5.4. Non-Human Adenovirus Vectored H5N1 Vaccines

Several non-human Ad vectors have been tested as H5N1 vaccine carriers that were primarily intended as strategies to overcome pre-existing anti-Ad5 immunity. Roy *et al.* tested the efficacy of a single dose of AdC7 (a chimpanzee adenovirus vector) expressing A/Puerto Rico/8/34/mount Sinai NP gene and showed that it was less effective than a Ad5 based vector in protection against A/Puerto Rico/8/34/mount Sinai, A/Vitetnam/1203/04 and A/Hong Kong/483/1997 in a mouse model [87]. Singh *et al.* described the effectiveness of a BAd3 (a bovine adenovirus 3 vector) containing HA gene from A/Hong Kong/156/97 in eliciting the protective immune responses against A/Hong Kong/483/97 challenges in mice with or without pre-existing anti-Ad5 immunity [88]. Patel *et al.* presented data with a PAV3 (a porcine adenovirus 3 vector) based H5N1 vaccine encoding A/Hanoi/30408/05 HA gene and demonstrated that it could be at least as potent as its Ad5 counterpart in mice [90]. A recombinant replication-competent canine adenovirus type 2 (cAd2) expressing HA gene from H5N1 subtype of tiger influenza virus (A/tiger/Harbin/01/2003) has also been evaluated in cats as a Felidae H5N1 vaccine [164].

### 5.5. Recombinant Adenoviral Vectored H5N1 in Chicken

Most H5N1 infections in humans have been preceded by outbreaks in poultry [165]. Thus, it is not surprising that mass immunization of poultry against H5N1 could reduce the risk of human exposure; hence, the decrease of H5N1 infected humans by reducing the number of susceptible poultry [166]. Current vaccination of poultry with oil emulsion, inactivated, whole-AI-virus vaccines or fowl pox-vectored AI vaccines are effective in reducing the risk of infection and disease, but injection of individual birds are labor intensive and time consuming. Improved vaccines are required in order to induce rapid and sustained broad immunity, to allow mass vaccination campaigns and DIVA (differentiation between infected and vaccinated animals) strategies. The potency of a rAd vectored H5N1 vaccine expressing HA protein of A/Vietnam/1203/04 has been demonstrated [101,118] and can be significantly enhanced by fusion of HA protein with chicken CD154 in chickens [167]. Additionally, chickens can be effectively immunized by in *ovo* administration or aerosol spray of rAd vectored AI vaccines [125,168]. Both types of vaccines may allow mass immunization of poultry with the rAd vectored vaccines. Furthermore, chickens immunized in ovo with a rAd vectored HA5 vaccine can be successfully vaccinated post-hatch with another rAd vectored HA7 vaccine [122], raising the possibility of protection against multiple pathogens with the same rAd vector technology.

## 6. Future Directions

There is no doubt that significant progress has been made, during the past decade, in the field of the rAd-vectored H5N1 influenza vaccines. However, these vaccines must overcome several challenges before they can be considered a suitable alternative to the currently licensed vaccines. 

A major drawback faced by the vaccine candidates for licensure is the lack of information associated to the correlation between immunity and protection [169]. A serum HI titer of 40 or greater is a well-established marker of immune protection for inactivated seasonal influenza vaccines intramuscularly injected. However, this may not appear to hold true for a rAd vectored H5N1 vaccine encoding heterogonous HA [128,150], HA1 fragment (Vaxin unpublished data), HA2 fragment [118], or conserved NP and M2 antigens [129]. Mounting evidence indicates that mucosal and T cell mediated immunity may actually more important than previously realized against a broad spectrum of H5N1 strains [118,119,121,123,128,129]. Therefore, the standardization of immunoassays used in the assessment of the innate and adaptive immune responses is crucial for the comparative analysis of such vaccines. Also, it is important to standardize virulent H5N1 challenge reagents and animal models for head-to-head comparisons of rAd H5N1 vaccine candidates.

The cross-protection achieved by vaccination with rAd vaccines encoding HA and/or conserved antigens is very encouraging [52,87,129,147]. In a pandemic scenario, these vaccines may be used for emergency vaccination when an antigenically matched vaccine is not available [170]. Future nonclinical and clinical studies should be aimed to identify the optimal combination of rAd expressing HA and/or conserved NP, M1, M2 antigens for the induction of protective immunity against heterogonous strains.

The development of rAd vectored H5N1 vaccines is greatly benefited from advanced synthetic DNA technologies. With improvements in surveillance and case confirmation, the modified HA or other antigen can be rapidly synthesized in order to adjust the changes in current H5N1 strains. Nonclinical studies showed that rAd or DNA vaccines encoding synthetic consensus HA genes are effective for eliciting protective immunity against mismatched virus challenges [140,171,172,173]. The immune responses could be substantially strengthened with codon-optimized influenza antigens inserted into rAd vectored vaccines [148,174]. However, our previous experience with manufacturing of rAd vaccines encoding codon-optimized HA genes showed that overexpression of HA transgene products from a number of influenza virus strains may inhibit rAd vector production in replication permissive cell lines (Vaxin unpublished data). This suggests that manufacturing process optimization will be required to maximize yields prior to scale up. 

Until recently, it was believed that the common presence of pre-existing Ad5 immunity in human populations could be a potential problem for the clinical use of rAd vectored vaccines. Although not studied in sufficient detail, emerging data from clinical trials suggest that this limitation can be overcome by increasing the vaccine dose [175] or by intranasal vaccination [53]. However, more clinical information is required to clarify the influence of the pre-existing immunity on the rAd vectored vaccines.

Vaxin’s nonclinical and Phase I clinical trial data support the overall advantages of our Ad5-vectored nasal influenza vaccine platform [53] (Vaxin unpublished data). These data are moving forward to more advanced clinical stages. Further evaluation of rAd vectored seasonal and pandemic influenza vaccines in animal models and clinical trials is needed to confirm their suitability for human use. They must include the in high-risk populations, such as the very young, the elderly, pregnant women, and immunocompromised individuals. 

Influenza vaccines based on egg-independent technologies have increased acceptance in recent years. Mammalian cell-based vaccines have been licensed by regulatory agencies in Europe, Asia, and Latin America [176] and close to commercialization in the USA [177]. Recombinant protein-based vaccines and VLP based vaccines can be produced in plants, insect cells, or *Escherichia coli *[178]. VLP based vaccines can induce both humoral and cellular immunity in nonclinical studies and have looked very promising in clinical trials [178]. rAd vectored H5N1 vaccines are considered lead-candidates among DNA based and viral vectored influenza vaccines [35]. Based on the promising results of rAd H5N1 vaccine in nonclinical and clinical studies and the increasing clinical experience with rAd vectored vaccines against various infectious pathogens, we believe that the rAd vectored nasal influenza vaccines hold great promise for the influenza pandemic preparedness.

## References

[1] Thompson W.W., Shay D.K., Weintraub E., Brammer L., Bridges C.B., Cox N.J., Fukuda K. (2004). Influenza-associated hospitalizations in the United States. JAMA.

[2] Centers for Disease Control and Prevention (CDC) (2010). Estimates of deaths associated with seasonal influenza: United States, 1976-2007. MMWR Morb. Mortal. Wkly. Rep..

[3] Pérez Velasco R., Praditsitthikorn N., Wichmann K., Mohara A., Kotirum S., Tantivess S., Vallenas C., Harmanci H., Teerawattananon Y. (2012). Systematic review of economic evaluations of preparedness strategies and interventions against influenza pandemics. PLoS One.

[4] Lagacé-Wiens P.R., Rubinstein E., Gumel A. (2010). Influenza epidemiology: Past, present, and future. Crit. Care Med..

[5] Fouchier R.A., Munster V., Wallensten A., Bestebroer T.M., Herfst S., Smith D., Rimmelzwaan G.F., Olsen B., Osterhaus A.D. (2005). Characterization of a novel influenza A virus hemagglutinin subtype (H16) obtained from black-headed gulls. J. Virol..

[6] Tong S., Li Y., Rivailler P., Conrardy C., Castillo D.A., Chen L.M., Recuenco S., Ellison J.A., Davis C.T., York I.A., Turmelle A.S., Moran D., Rogers. S., Shi M., Tao Y., Weil M.R., Tang K., Rowe L.A., Sammons S., Xu X., Frace M., Lindblade K.A., Cox N.J., Anderson L.J., Rupprecht C.E., Donis R.O. (2012). A distinct lineage of influenza A virus from bats. Proc. Natl. Acad. Sci. USA.

[7] Scholtissek C., Rohde W., Von Hoyningen V., Rott R. (1978). On the origin of the human influenza virus subtypes H2N2 and H3N2. Virology.

[8] Reid A.H., Fanning T.G., Hultin J.V., Taubenberger J.K. (1999). Origin and evolution of the 1918 "Spanish" influenza virus hemagglutinin gene. Proc. Natl. Acad. Sci. USA.

[9] Trifonov V., Khiabanian H., Rabadan R. (2009). Geographic dependence, surveillance, and origins of the 2009 influenza A (H1N1) virus. N. Engl. J. Med..

[10] World Health Organization (WHO) (2012). Antigenic and genetic characteristics of zoonotic influenza viruses and development of candidate vaccine viruses for pandemic preparedness. Wkly. Epidemiol. Rec..

[11] Centers for Disease Control and Prevention (CDC) (1998). From the Centers for Disease Control and Prevention. Update: Isolation of avian influenza A(H5N1) viruses from humans: Hong Kong, 1997-1998. JAMA.

[12] Health Protection Agency (1997). Influenza A virus subtype H5N1 infection in humans: Update. Commun. Dis. Rep. CDR Wkly..

[13] Adams S., Sandrock C. (2010). Avian influenza: Update. Med. Princ. Pract..

[14] World Health Organization (WHO) 10 August 2012 update. Cumulative number of confirmed human cases for avian influenza A(H5N1) reported to WHO, 2003-2012. http://www.who.int/influenza/human_animal_interface/EN_GIP_20120502CumulativeNumberH5N1cases.pdf.

[15] Dudley J.P. (2012). Mammalian-transmissible highly pathogenic H5N1 influenza: Epidemiological context. MBio..

[16] Herfst S., Schrauwen E.J., Linster M., Chutinimitkul S., de Wit E., Munster V.J., Sorrell E.M., Bestebroer T.M., Burke D.F., Smith D.J., Rimmelzwaan G.F., Osterhaus A.D., Fouchier R.A. (2012). Airborne transmission of influenza A/H5N1 virus between ferrets. Science.

[17] Imai M., Watanabe T., Hatta M., Das S.C., Ozawa M., Shinya K., Zhong G., Hanson A., Katsura H., Watanabe S., Li C., Kawakami E., Yamada S., Kiso M., Suzuki Y., Maher E.A., Neumann G., Kawaoka Y. (2012). Experimental adaptation of an influenza H5 HA confers respiratory droplet transmission to a reassortant H5 HA/H1N1 virus in ferrets. Nature.

[18] Russell C.A., Fonville J.M., Brown A.E., Burke D.F., Smith D.L., James S.L., Herfst S., van Boheemen S., Linster M., Schrauwen E.J., Katzelnick L., Mosterín A., Kuiken T., Maher E., Neumann G., Osterhaus A.D., Kawaoka Y., Fouchier R.A., Smith D.J. (2012). The potential for respiratory droplet-transmissible A/H5N1 influenza virus to evolve in a mammalian host. Science.

[19] Webster R.G. (2012). Mammalian-transmissible H5N1 influenza: the dilemma of dual-use research. MBio..

[20] World Health Organization (WHO) Pandemic influenza preparedness and response: A WHO guidance document. http://whqlibdoc.who.int/publications/2009/9789241547680_eng.pdf.

[21] Lee V.J., Lye D.C., Wilder-Smith A. (2009). Combination strategies for pandemic influenza response - A systematic review of mathematical modeling studies. BMC Med..

[22] Schuchat A., Bell B.P., Redd S.C. (2011). The science behind preparing and responding to pandemic influenza: the lessons and limits of science. Clin. Infect. Dis..

[23] Emanuel E.J., Wertheimer A. (2006). Public health. Who should get influenza vaccine when not all can?. Science.

[24] Smith K.A., Colvin C.J., Weber P.S., Spatz S.J., Coussens P.M. (2008). High titer growth of human and avian influenza viruses in an immortalized chick embryo cell line without the need for exogenous proteases. Vaccine.

[25] Rockman S., Brown L. (2010). Pre-pandemic and pandemic influenza vaccines. Hum. Vaccin..

[26] Clayville L.R. (2011). Influenza update: A review of currently available vaccines. Pharmacy and Therapeutics.

[27] Centers for Disease Control and Prevention (CDC). http://www.cdc.gov/vaccines/pubs/vis/downloads/vis-flulive.pdf.

[28] Haaheim L.R., Madhun A.S., Cox R. (2009). Pandemic influenza vaccines - The challenges. Viruses.

[29] Singh N., Pandey A., Mittal S.K. (2010). Avian influenza pandemic preparedness: Developing prepandemic and pandemic vaccines against a moving target. Expert Rev. Mol. Med..

[30] Horimoto T., Kawaoka Y. (2009). Designing vaccines for pandemic influenza. Curr. Top. Microbiol. Immunol..

[31] Tutykhina I.L., Logunov D.Y., Shcherbinin D.N., Shmarov M.M., Tukhvatulin A.I., Naroditsky B.S., Gintsburg A.L. (2011). Development of adenoviral vector-based mucosal vaccine against influenza. J. Mol. Med. (Berl)..

[32] Tang D.C., Zhang J., Toro H., Shi Z., Van Kampen K.R. (2009). Adenovirus as a carrier for the development of influenza virus-free avian influenza vaccines. Expert Rev. Vaccines..

[33] Vemula S.V., Mittal S.K. (2010). Production of adenovirus vectors and their use as a delivery system for influenza vaccines. Expert. Opin. Biol. Ther..

[34] Toro H., Tang D.C. (2009). Protection of chickens against avian influenza with nonreplicating adenovirus-vectored vaccine. Poult. Sci..

[35] Lambe T. (2012). Novel viral vectored vaccines for the prevention of influenza. Mol. Med..

[36] Rowe W.P., Huebner R.J., Gilmore L.K., Parrott R.H., Ward T.G. (1953). Isolation of a cytopathogenic agent from human adenoids undergoing spontaneous degeneration in tissue culture. Proc. Soc. Exp. Biol. Med..

[37] Bangari D.S., Mittal S.K. (2006). Development of nonhuman adenoviruses as vaccine vectors. Vaccine..

[38] Smith J.G., Wiethoff C.M., Stewart P.L., Nemerow G.R. (2010). Adenovirus. Curr. Top. Microbiol. Immunol..

[39] Lichtenstein D.L., Wold W.S. (2004). Experimental infections of humans with wild-type adenoviruses and with replication-competent adenovirus vectors: replication, safety, and transmission. Cancer Gene Ther..

[40] Reddy V.S., Natchiar S.K., Stewart P.L., Nemerow G.R. (2010). Crystal structure of human adenovirus at 3.5 A resolution. Science.

[41] Ginsberg H.S. (1999). The life and times of adenoviruses. Adv. Virus. Res..

[42] Russell W.C. (2009). Adenoviruses: Update on structure and function. J. Gen. Virol..

[43] Young C.S. (2003). The structure and function of the adenovirus major late promoter. Curr. Top. Microbiol. Immunol..

[44] Graham F.L., Smiley J., Russell W.C., Nairn R. (1977). Characteristics of a human cell line transformed by DNA from human adenovirus type 5. J. Gen.Virol..

[45] Kovesdi I., Hedley S.J. (2010). Adenoviral producer cells. Viruses.

[46] Evans R.K, Nawrocki D.K., Isopi L.A., Williams D.M., Casimiro D.R., Chin S., Chen M., Zhu D.M., Shiver J.W., Volkin D.B. (2004). Development of stable liquid formulations for adenovirus-based vaccines. J. Pharm. Sci..

[47] Croyle M.A., Cheng X., Wilson J.M. (2001). Development of formulations that enhance physical stability of viral vectors for gene therapy. Gene. Ther..

[48] Alcock R., Cottingham M.G., Rollier C.S., Furze J., De Costa S.D., Hanlon M., Spencer A.J., Honeycutt J.D., Wyllie D.H., Gilbert S.C., Bregu M., Hill A.V. (2010). Long-term thermostabilization of live poxviral and adenoviral vaccine vectors at supraphysiological temperatures in carbohydrate glass. Sci. Transl. Med..

[49] Lewis D.J., Huo Z., Barnett S., Kromann I., Giemza R., Galiza E., Woodrow M., Thierry-Carstensen B., Andersen P., Novicki D., Del Giudice G., Rappuoli R. (2009). Transient facial nerve paralysis (Bell's palsy) following intranasal delivery of a genetically detoxified mutant of Escherichia coli heat labile toxin. PLoS One.

[50] Molinier-Frenkel V., Lengagne R., Gaden F., Hong S.S., Choppin J., Gahery-Ségard H., Boulanger P., Guillet J.G. (2002). Adenovirus hexon protein is a potent adjuvant for activation of a cellular immune response. J. Virol..

[51] Hartman Z.C., Appledorn D.M., Amalfitano A. (2008). Adenovirus vector induced innate immune responses: Impact upon efficacy and toxicity in gene therapy and vaccine applications. Virus Res..

[52] Wei C.J., Boyington J.C., McTamney P.M., Kong W.P., Pearce M.B., Xu L., Andersen H., Rao S., Tumpey T.M., Yang Z.Y., Nabel G.J. (2010). Induction of broadly neutralizing H1N1 influenza antibodies by vaccination. Science.

[53] Van Kampen K.R., Shi Z.., Gao P., Zhang J., Foster K.W., Chen D.T., Marks D., Elmets C.A., Tang D.C. (2005). Safety and immunogenicity of adenovirus-vectored nasal and epicutaneous influenza vaccines in humans. Vaccine.

[54] Hashem A., Jaentschke B., Gravel C., Tocchi M., Doyle T., Rosu-Myles M., He R., Li X. (2012). Subcutaneous immunization with recombinant adenovirus expressing influenza A nucleoprotein protects mice against lethal viral challenge. Hum. Vaccin. Immunother..

[55] Song K., Bolton D.L., Wei C.J., Wilson R.L., Camp J.V., Bao S., Mattapallil J.J., Herzenberg L.A., Herzenberg L.A., Andrews C.A., Sadoff J.C., Goudsmit J., Pau M.G., Seder R.A., Kozlowski P.A., Nabel G.J., Roederer M., Rao S.S. (2010). Genetic immunization in the lung induces potent local and systemic immune responses. Proc. Natl. Acad. Sci. USA.

[56] Roy C.J., Ault A., Sivasubramani S.K., Gorres J.P., Wei C.J., Andersen H., Gall J., Roederer M., Rao S.S. (2011). Aerosolized adenovirus-vectored vaccine as an alternative vaccine delivery method. Respir. Res..

[57] Saito I., Oya Y., Yamamoto K., Yuasa T., Shimojo H. (1985). Construction of nondefective adenovirus type 5 bearing a 2.8-kilobase hepatitis B virus DNA near the right end of its genome. J. Virol..

[58] Morin J.E., Lubeck M.D., Barton J.E., Conley A.J., Davis A.R., Hung P.P. (1987). Recombinant adenovirus induces antibody response to hepatitis B virus surface antigen in hamsters. Proc. Natl. Acad. Sci. USA.

[59] Alkhatib G., Briedis D.J. (1988). High-level eucaryotic in vivo expression of biologically active measles virus hemagglutinin by using an adenovirus type 5 helper-free vector system. J. Virol..

[60] Eloit M., Gilardi-Hebenstreit P., Toma B., Perricaudet M. (1990). Construction of a defective adenovirus vector expressing the pseudorabies virus glycoprotein gp50 and its use as a live vaccine. J. Gen. Virol..

[61] Zhu J., Grace M., Casale J., Chang A.T., Musco M.L., Bordens R., Greenberg R., Schaefer E., Indelicato S.R. (1999). Characterization of replication-competent adenovirus isolates from large-scale production of a recombinant adenoviral vector. Hum. Gene. Ther..

[62] Lochmüller H., Jani A., Huard J., Prescott S., Simoneau M., Massie B., Karpati G., Acsadi G. (1994). Emergence of early region 1-containing replication-competent adenovirus in stocks of replication-defective adenovirus recombinants (delta E1 + delta E3) during multiple passages in 293 cells. Hum. Gene. Ther..

[63] Hermens W.T., Verhaagen J. (1997). Adenoviral vector-mediated gene expression in the nervous system of immunocompetent Wistar and T cell-deficient nude rats: preferential survival of transduced astroglial cells in nude rats. Hum. Gene. Ther..

[64] Fallaux F.J., Bout A., van der Velde I., van den Wollenberg D.J., Hehir K.M., Keegan J., Auger C., Cramer S.J., van Ormondt H., van der Eb A.J., Valerio D., Hoeben R.C. (1998). New helper cells and matched early region 1-deleted adenovirus vectors prevent generation of replication-competent adenoviruses. Hum. Gene. Ther..

[65] Ledwith B.J., Lanning C.L., Gumprecht L.A, Anderson C.A., Coleman J.B., Gatto N.T., Balasubramanian G., Farris G.M., Kemp R.K., Harper L.B., Barnum A.B., Pacchione S.J., Mauer K.L., Troilo P.F., Brown E.R., Wolf J.J., Lebronl J.A., Lewis J.A., Nichols W.W. (2006). Tumorigenicity assessments of Per.C6 cells and of an Ad5-vectored HIV-1 vaccine produced on this continuous cell line. Dev. Biol. (Basel).

[66] Lusky M. (2005). Good manufacturing practice production of adenoviral vectors for clinical trials. Hum Gene Ther..

[67] Subramanian S., Kim J.J., Harding F., Altaras G.M., Aunins J.G., Zhou W. (2007). Scaleable production of adenoviral vectors by transfection of adherent PER.C6 cells. Biotechnol. Prog..

[68] Wang Q., Jia X.C., Finer M.H. (1995). A packaging cell line for propagation of recombinant adenovirus vectors containing two lethal gene-region deletions. Gene. Ther..

[69] Amalfitano A., Begy C.R., Chamberlain J.S. (1996). Improved adenovirus packaging cell lines to support the growth of replication-defective gene-delivery vectors. Proc. Natl. Acad. Sci. USA.

[70] Gorziglia M.I., Kadan M.J., Yei S., Lim J., Lee G.M., Luthra R., Trapnell B.C. (1996). Elimination of both E1 and E2 from adenovirus vectors further improves prospects for *in vivo* human gene therapy. J. Virol..

[71] Gorziglia M.I., Lapcevich C., Roy S., Kang Q., Kadan M., Wu V., Pechan P., Kaleko M. (1999). Generation of an adenovirus vector lacking E1, e2a, E3, and all of E4 except open reading frame 3. J. Virol..

[72] Mitani K., Graham F.L., Caskey C.T., Kochanek S. (1995). Rescue, propagation, and partial purification of a helper virus-dependent adenovirus vector. Proc. Natl. Acad. Sci. USA.

[73] Osada T., Yang X.Y., Hartman Z.C., Glass O., Hodges B.L., Niedzwiecki D., Morse M.A., Lyerly H.K., Amalfitano A., Clay T.M. (2009). Optimization of vaccine responses with an E1, E2b and E3-deleted Ad5 vector circumvents pre-existing anti-vector immunity. Cancer Gene. Ther..

[74] Weaver E.A., Nehete P.N., Buchl S.S., Senac J.S., Palmer D., Ng P., Sastry K.J., Barry M.A. (2009). Comparison of replication-competent, first generation, and helper-dependent adenoviral vaccines. PLoS One.

[75] Mast T.C., Kierstead L., Gupta S.B., Nikas A.A., Kallas E.G., Novitsky V., Mbewe B., Pitisuttithum P., Schechter M., Vardas E., Wolfe N.D., Aste-Amezaga M., Casimiro D.R., Coplan P., Straus W.L., Shiver J.W. (2010). International epidemiology of human pre-existing adenovirus (Ad) type-5, type-6, type-26 and type-36 neutralizing antibodies: correlates of high Ad5 titers and implications for potential HIV vaccine trials. Vaccine.

[76] Barouch D.H., Kik S.V., Weverling G.J., Dilan R., King S.L., Maxfield L.F., Clark S., Ng'ang'a D., Brandariz K.L., Abbink P., Sinangil F., de Bruyn G., Gray G.E., Roux S., Bekker L.G., Dilraj A., Kibuuka H., Robb M.L., Michael N.L., Anzala O., Amornkul P.N., Gilmour J., Hural J., Buchbinder S.P., Seaman M.S., Dolin R., Baden L.R., Carville A., Mansfield K.G., Pau M.G., Goudsmit J. (2011). International seroepidemiology of adenovirus serotypes 5, 26, 35, and 48 in pediatric and adult populations. Vaccine.

[77] Barouch D.H., McKay P.F., Sumida S.M., Santra S., Jackson S.S., Gorgone D.A., Lifton M.A., Chakrabarti B.K., Xu L., Nabel G.J., Letvin N.L. (2003). Plasmid chemokines and colony-stimulating factors enhance the immunogenicity of DNA priming-viral vector boosting human immunodeficiency virus type 1 vaccines. J. Virol..

[78] Pichla-Gollon S.L., Lin S.W., Hensley S.E., Lasaro M.O., Herkenhoff-Haut L., Drinker M., Tatsis N., Gao G.P., Wilson J.M., Ertl H.C., Bergelson J.M. (2009). Effect of preexisting immunity on an adenovirus vaccine vector: in vitro neutralization assays fail to predict inhibition by antiviral antibody in vivo. J. Virol..

[79] Yang Z.Y., Wyatt L.S., Kong W.P., Moodie Z., Moss B., Nabel G.J. (2003). Overcoming immunity to a viral vaccine by DNA priming before vector boosting. J. Virol..

[80] Vogels R., Zuijdgeest D., van Rijnsoever R., Hartkoorn E., Damen I., de Béthune M.P., Kostense S., Penders G., Helmus N., Koudstaal W., Cecchini M., Wetterwald A., Sprangers M., Lemckert A., Ophorst O., Koel B., van Meerendonk M., Quax P., Panitti L., Grimbergen J., Bout A., Goudsmit J., Havenga M. (2003). Replication-deficient human adenovirus type 35 vectors for gene transfer and vaccination: Efficient human cell infection and bypass of preexisting adenovirus immunity. J. Virol..

[81] Holterman L., Vogels R., van der Vlugt R., Sieuwerts M., Grimbergen J., Kaspers J., Geelen E., van der Helm E., Lemckert A., Gillissen G., Verhaagh S., Custers J., Zuijdgeest D., Berkhout B., Bakker M., Quax P., Goudsmit J., Havenga M. (2004). Novel replication-incompetent vector derived from adenovirus type 11 (Ad11) for vaccination and gene therapy: Low seroprevalence and non-cross-reactivity with Ad5. J. Virol..

[82] Lemckert A.A., Grimbergen J., Smits S., Hartkoorn E., Holterman L., Berkhout B., Barouch D.H., Vogels R., Quax P., Goudsmit J., Havenga M.J. (2006). Generation of a novel replication-incompetent adenoviral vector derived from human adenovirus type 49: manufacture on PER.C6 cells, tropism and immunogenicity. J. Gen. Virol..

[83] Abbink P., Lemckert A.A., Ewald B.A., Lynch D.M., Denholtz M., Smits S., Holterman L., Damen I., Vogels R., Thorner A.R., O'Brien K.L., Carville A., Mansfield K.G., Goudsmit J., Havenga M.J., Barouch D.H. (2007). Comparative seroprevalence and immunogenicity of six rare serotype recombinant adenovirus vaccine vectors from subgroups B and D. J. Virol..

[84] Kahl C.A., Bonnell J., Hiriyanna S., Fultz M., Nyberg-Hoffman C., Chen P., King C.R., Gall J.G. (2010). Potent immune responses and in vitro pro-inflammatory cytokine suppression by a novel adenovirus vaccine vector based on rare human serotype 28. Vaccine.

[85] Tatsis N., Tesema L., Robinson E.R., Giles-Davis W., McCoy K., Gao G.P., Wilson J.M., Ertl H.C. (2006). Chimpanzee-origin adenovirus vectors as vaccine carriers. Gene. Ther..

[86] Xiang Z., Gao G., Reyes-Sandoval A., Cohen C.J., Li Y., Bergelson J.M., Wilson J.M., Ertl H.C. (2002). Novel, chimpanzee serotype 68-based adenoviral vaccine carrier for induction of antibodies to a transgene product. J. Virol..

[87] Roy S., Kobinger G.P., Lin J., Figueredo J., Calcedo R., Kobasa D., Wilson J.M. (2007). Partial protection against H5N1 influenza in mice with a single dose of a chimpanzee adenovirus vector expressing nucleoprotein. Vaccine.

[88] Singh N., Pandey A., Jayashankar L., Mittal S.K. (2008). Bovine adenoviral vector-based H5N1 influenza vaccine overcomes exceptionally high levels of pre-existing immunity against human adenovirus. Mol. Ther..

[89] Gao Y.W., Xia X.Z., Wang L.G., Liu D., Huang G. (2006). Construction and experimental immunity of recombinant replication-competent canine adenovirus type 2 expressing hemagglutinin gene of H5N1 subtype tiger influenza virus. Wei Sheng Wu Xue Bao.

[90] Patel A., Tikoo S., Kobinger G. (2010). A porcine adenovirus with low human seroprevalence is a promising alternative vaccine vector to human adenovirus 5 in an H5N1 virus disease model. PLoS One.

[91] Wang L., Cheng C., Ko S.Y., Kong W.P., Kanekiyo M., Einfeld D., Schwartz R.M., King C.R., Gall J.G., Nabel G.J. (2009). Delivery of human immunodeficiency virus vaccine vectors to the intestine induces enhanced mucosal cellular immunity. J. Virol..

[92] Chen H., Xiang Z.Q., Li Y., Kurupati R.K., Jia B., Bian A., Zhou D.M., Hutnick N., Yuan S., Gray C., Serwanga J., Auma B., Kaleebu P., Zhou X., Betts M.R., Ertl H.C. (2010). Adenovirus-based vaccines: Comparison of vectors from three species of adenoviridae. J. Virol..

[93] Fitzgerald J.C., Gao G.P., Reyes-Sandoval A., Pavlakis G.N., Xiang Z.Q., Wlazlo A.P., Giles-Davis W., Wilson J.M., Ertl H.C. (2003). A simian replication-defective adenoviral recombinant vaccine to HIV-1 gag. .J. Immunol..

[94] Frahm N., DeCamp A.C., Friedrich D.P., Carter D.K., Defawe O.D., Kublin J.G., Casimiro D.R., Duerr A., Robertson M.N., Buchbinder S.P., Huang Y., Spies G.A., De Rosa S.C., McElrath M.J. (2012). Human adenovirus-specific T cells modulate HIV-specific T cell responses to an Ad5-vectored HIV-1 vaccine. J. Clin. Invest..

[95] Seregin S.S., Amalfitano A. (2009). Overcoming pre-existing adenovirus immunity by genetic engineering of adenovirus-based vectors. Expert Opin. Biol. Ther..

[96] Dharmapuri S., Peruzzi D., Aurisicchio L. (2009). Engineered adenovirus serotypes for overcoming anti-vector immunity. Expert Opin. Biol. Ther..

[97] Stone D., Liu Y., Li Z.Y., Tuve S., Strauss R., Lieber A. (2007). Comparison of adenoviruses from species B, C, E, and F after intravenous delivery. Mol. Ther..

[98] Holst P.J., Ørskov C., Thomsen A.R., Christensen J.P. (2010). Quality of the transgene-specific CD8+ T cell response induced by adenoviral vector immunization is critically influenced by virus dose and route of vaccination. J. Immunol..

[99] Kaufman D.R., Bivas-Benita M., Simmons N.L., Miller D., Barouch D.H. (2010). Route of adenovirus-based HIV-1 vaccine delivery impacts the phenotype and trafficking of vaccine-elicited CD8+ T lymphocytes. J. Virol..

[100] Suda T., Kawano M., Nogi Y., Ohno N., Akatsuka T., Matsui M. (2011). The route of immunization with adenoviral vaccine influences the recruitment of cytotoxic T lymphocytes in the lung that provide potent protection from influenza A virus. Antiviral Res..

[101] Steitz J., Wagner R.A., Bristol T., Gao W., Donis R.O., Gambotto A. (2010). Assessment of route of administration and dose escalation for an adenovirus-based influenza A Virus (H5N1) vaccine in chickens. Clin. Vaccine. Immunol..

[102] Holman D.H., Penn-Nicholson A., Wang D., Woraratanadharm J., Harr M.K., Luo M., Maher E.M., Holbrook M.R., Dong J.Y. (2009). A complex adenovirus-vectored vaccine against Rift Valley fever virus protects mice against lethal infection in the presence of preexisting vector immunity. Clin. Vaccine Immunol..

[103] Appledorn D.M., Aldhamen Y.A., Godbehere S., Seregin S.S., Amalfitano A. (2011). Sublingual administration of an adenovirus serotype 5 (Ad5)-based vaccine confirms Toll-like receptor agonist activity in the oral cavity and elicits improved mucosal and systemic cell-mediated responses against HIV antigens despite preexisting Ad5 immunity. Clin. Vaccine Immunol..

[104] Xiang Z.Q., Gao G.P., Reyes-Sandoval A., Li Y., Wilson J.M., Ertl H.C. (2003). Oral vaccination of mice with adenoviral vectors is not impaired by preexisting immunity to the vaccine carrier. J. Virol..

[105] Pandey A., Singh N., Vemula S.V., Couëtil L., Katz J.M., Donis R., Sambhara S., Mittal S.K. (2012). Impact of preexisting adenovirus vector immunity on immunogenicity and protection conferred with an adenovirus-based H5N1 influenza vaccine. PLoS One.

[106] Xiang Z.Q., Yang Y., Wilson J.M., Ertl H.C. (1996). A replication-defective human adenovirus recombinant serves as a highly efficacious vaccine carrier. Virology.

[107] Shi Z., Zeng M., Yang G., Siegel F., Cain L.J., van Kampen K.R., Elmets C.A., Tang D.C. (2001). Protection against tetanus by needle-free inoculation of adenovirus-vectored nasal and epicutaneous vaccines. J. Virol..

[108] Yu J.R., Kim S., Lee J.B., Chang J. (2008). Single intranasal immunization with recombinant adenovirus-based vaccine induces protective immunity against respiratory syncytial virus infection. J. Virol..

[109] Croyle M.A., Patel A., Tran K.N., Gray M., Zhang Y., Strong J.E., Feldmann H., Kobinger G.P. (2008). Nasal delivery of an adenovirus-based vaccine bypasses pre-existing immunity to the vaccine carrier and improves the immune response in mice. PLoS One.

[110] Xu Q., Pichichero M.E., Simpson L.L., Elias M., Smith L.A., Zeng M. (2009). An adenoviral vector-based mucosal vaccine is effective in protection against botulism. Gene Ther..

[111] Richardson J.S., Abou M.C., Tran K.N., Kumar A., Sahai B.M., Kobinger G.P. (2011). Impact of systemic or mucosal immunity to adenovirus on Ad-based Ebola virus vaccine efficacy in guinea pigs. J. Infect. Dis..

[112] Draghia R., Caillaud C., Manicom R., Pavirani A., Kahn A., Poenaru L. (1995). Gene delivery into the central nervous system by nasal instillation in rats. Gene Ther..

[113] Damjanovic D., Zhang X., Mu J., Medina M.F., Xing Z. (2008). Organ distribution of transgene expression following intranasal mucosal delivery of recombinant replication-defective adenovirus gene transfer vector. Genet. Vaccines Ther..

[114] Huang D., Pereboev A.V., Korokhov N., He R., Larocque L., Gravel C., Jaentschke B., Tocchi M., Casley W.L., Lemieux M., Curiel D.T., Chen W., Li X. (2008). Significant alterations of biodistribution and immune responses in Balb/c mice administered with adenovirus targeted to CD40(+) cells. Gene Ther..

[115] Lemiale F., Kong W.P., Akyürek L.M., Ling X., Huang Y., Chakrabarti B.K., Eckhaus M., Nabel G.J. (2003). Enhanced mucosal immunoglobulin A response of intranasal adenoviral vector human immunodeficiency virus vaccine and localization in the central nervous system. J. Virol..

[116] Näslund T.I., Uyttenhove C., Nordström E.K., Colau D., Warnier G., Jondal M., Van den Eynde B.J., Liljeström P. (2007). Comparative prime-boost vaccinations using Semliki Forest virus, adenovirus, and ALVAC vectors demonstrate differences in the generation of a protective central memory CTL response against the P815 tumor. J. Immunol..

[117] Barefoot B., Thornburg N.J., Barouch D.H., Yu J.S., Sample C., Johnston R.E., Liao H.X., Kepler T.B., Haynes B.F., Ramsburg E. (2008). Comparison of multiple vaccine vectors in a single heterologous prime-boost trial. Vaccine.

[118] Gao W., Soloff A.C., Lu X., Montecalvo A., Nguyen D.C., Matsuoka Y., Robbins P.D., Swayne D.E., Donis R.O., Katz J.M., Barratt-Boyes S.M., Gambotto A. (2006). Protection of mice and poultry from lethal H5N1 avian influenza virus through adenovirus-based immunization. J. Virol..

[119] Hoelscher M.A., Garg S., Bangari D.S., Belser J.A., Lu X., Stephenson I., Bright R.A., Katz J.M., Mittal S.K., Sambhara S. (2006). Development of adenoviral-vector-based pandemic influenza vaccine against antigenically distinct human H5N1 strains in mice. Lancet.

[120] Thomas P.G., Keating R., Hulse-Post D.J., Doherty P.C. (2006). Cell-mediated protection in influenza infection. Emerg. Infect. Dis..

[121] Lin J., Somanathan S., Roy. S., Calcedo R., Wilson J.M. (2010). Lung homing CTLs and their proliferation ability are important correlates of vaccine protection against influenza. Vaccine.

[122] Toro H., Tang D.C., Suarez D.L., Zhang J., Shi Z. (2008). Protection of chickens against avian influenza with non-replicating adenovirus-vectored vaccine. Vaccine.

[123] Shmarov M.M., Sedova E.S., Verkhovskaya L.V., Rudneva I.A, Bogacheva E.A., Barykova Y.A., Shcherbinin D.N., Lysenko A.A., Tutykhina I.L., Logunov D.Y., Smirnov Y.A., Naroditsky B.S., Gintsburg A.L. (2010). Induction of a protective heterosubtypic immune response against the Influenza virus by using recombinant adenoviral vectors expressing hemagglutinin of the Influenza H5 virus. Acta Naturae.

[124] Du L., Zhou Y., Jiang S. (2010). Research and development of universal influenza vaccines. Microbes Infect..

[125] Toro H., Tang D.C., Suarez D.L., Sylte M.J., Pfeiffer J., Van Kampen K.R. (2007). Protective avian influenza in ovo vaccination with non-replicating human adenovirus vector. Vaccine.

[126] Soboleski M.R., Gabbard J.D., Price G.E., Misplon J.A., Lo C.Y., Perez D.R., Ye J., Tompkins S.M., Epstein S.L. (2011). Cold-adapted influenza and recombinant adenovirus vaccines induce cross-protective immunity against pH1N1 challenge in mice. PLoS One.

[127] Price G.E., Soboleski M.R., Lo C.Y., Misplon J.A., Quirion M.R., Houser K.V., Pearce M.B., Pappas C., Tumpey T.M., Epstein S.L. (2010). Single-dose mucosal immunization with a candidate universal influenza vaccine provides rapid protection from virulent H5N1, H3N2 and H1N1 viruses. PLoS One.

[128] Park K.S., Lee J., Ahn S.S., Byun Y.H., Seong B.L., Baek Y.H., Song M.S., Choi Y.K., Na Y.J., Hwang I., Sung Y.C., Lee C.G. (2009). Mucosal immunity induced by adenovirus-based H5N1 HPAI vaccine confers protection against a lethal H5N2 avian influenza virus challenge. Virology.

[129] Price G.E., Soboleski M.R., Lo C.Y., Misplon J.A., Pappas C., Houser K.V., Tumpey T.M., Epstein S.L. (2009). Vaccination focusing immunity on conserved antigens protects mice and ferrets against virulent H1N1 and H5N1 influenza A viruses. Vaccine.

[130] Hasegawa H., Ichinohe T., Ainai A., Tamura S., Kurata T. (2009). Development of mucosal adjuvants for intranasal vaccine for H5N1 influenza viruses. Ther. Clin. Risk Manag..

[131] Tamura S., Tanimoto T., Kurata T. (2005). Mechanisms of broad cross-protection provided by influenza virus infection and their application to vaccines. Jpn. J. Infect. Dis..

[132] Ichinohe T., Tamura S., Kawaguchi A., Ninomiya A., Imai M., Itamura S., Odagiri T., Tashiro M., Takahashi H., Sawa H., Mitchell W.M., Strayer D.R., Carter W.A., Chiba J., Kurata T., Sata T., Hasegawa H. (2007). Cross-protection against H5N1 influenza virus infection is afforded by intranasal inoculation with seasonal trivalent inactivated influenza vaccine. J. Infect. Dis..

[133] Perrone L.A., Ahmad A., Veguilla V., Lu X., Smith G., Katz J.M., Pushko P., Tumpey T.M. (2009). Intranasal vaccination with 1918 influenza virus-like particles protects mice and ferrets from lethal 1918 and H5N1 influenza virus challenge. J. Virol..

[134] Lau Y.F., Wright A.R., Subbarao K. (2012). The contribution of systemic and pulmonary immune effectors to vaccine-induced protection from H5N1 influenza virus infection. J. Virol..

[135] Gustin K.M., Maines T.R., Belser J.A., van Hoeven N., Lu X., Dong L., Isakova-Sivak I., Chen L.M., Voeten J.T., Heldens J.G., van den Bosch H., Cox N.J., Tumpey T.M., Klimov A.I., Rudenko L., Donis R.O., Katz J.M. (2011). Comparative immunogenicity and cross-clade protective efficacy of mammalian cell-grown inactivated and live attenuated H5N1 reassortant vaccines in ferrets. J. Infect. Dis..

[136] Zhang J., Tarbet E.B., Feng T., Shi Z., Van Kampen K.R., Tang D.C. (2011). Adenovirus-vectored drug-vaccine duo as a rapid-response tool for conferring seamless protection against influenza. PLoS One.

[137] McConnell M.J., Hanna P.C., Imperiale M.J. (2007). Adenovirus-based prime-boost immunization for rapid vaccination against anthrax. Mol. Ther..

[138] Holst P.J., Bartholdy C., Stryhn A., Thomsen A.R., Christensen J.P. (2007). Rapid and sustained CD4(+) T-cell-independent immunity from adenovirus-encoded vaccine antigens. J. Gen. Virol..

[139] Fernández E., Toledo J.R., Chiong M., Parra F., Rodríguez E., Montero C., Méndez L., Capucci L., Farnós O. (2011). Single dose adenovirus vectored vaccine induces a potent and long-lasting immune response against rabbit hemorrhagic disease virus after parenteral or mucosal administration. Vet. Immunol. Immunopathol..

[140] Weaver E.A., Rubrum A.M., Webby R.J., Barry M.A. (2011). Protection against divergent influenza H1N1 virus by a centralized influenza hemagglutinin. PLoS One.

[141] Hoelscher M.A., Jayashankar L., Garg S., Veguilla V., Lu X., Singh N., Katz J.M., Mittal S.K., Sambhara S. (2007). New pre-pandemic influenza vaccines: an egg- and adjuvant-independent human adenoviral vector strategy induces long-lasting protective immune responses in mice. Clin. Pharmacol. Ther..

[142] Rao S.S., Kong W.P., Wei C.J., Van Hoeven N., Gorres J.P., Nason M., Andersen H., Tumpey T.M., Nabel G.J. (2010). Comparative efficacy of hemagglutinin, nucleoprotein, and matrix 2 protein gene-based vaccination against H5N1 influenza in mouse and ferret. PLoS One.

[143] Patel A., Tran K., Gray M., Li Y., Ao Z., Yao X., Kobasa D., Kobinger G.P. (2009). Evaluation of conserved and variable influenza antigens for immunization against different isolates of H5N1 viruses. Vaccine.

[144] Chen Q., Kuang H., Wang H., Fang F., Yang Z., Zhang Z., Zhang X., Chen Z. (2009). Comparing the ability of a series of viral protein-expressing plasmid DNAs to protect against H5N1 influenza virus. Virus Gene..

[145] Nayak B., Kumar S., DiNapoli J.M., Paldurai A., Perez D.R., Collins P.L., Samal S.K. (2010). Contributions of the avian influenza virus HA, NA, and M2 surface proteins to the induction of neutralizing antibodies and protective immunity. J. Virol..

[146] World Health Organisation (WHO), World Organisation for Animals Health United Nations (OIE), Food and Agriculture Organisation (FAO) H5N1 Evolution Working Group (2012). Continued evolution of highly pathogenic avian influenza A (H5N1): updated nomenclature. Influenza Other Respi. Viruses.

[147] Hoelscher M.A., Singh N., Garg S., Jayashankar L., Veguilla V., Pandey A., Matsuoka Y., Katz J.M., Donis R., Mittal S.K., Sambhara S. (2008). A broadly protective vaccine against globally dispersed clade 1 and clade 2 H5N1 influenza viruses. J. Infect. Dis..

[148] Hu X., Meng W., Dong Z., Pan W., Sun C., Chen L. (2011). Comparative immunogenicity of recombinant adenovirus-vectored vaccines expressing different forms of hemagglutinin (HA) proteins from the H5 serotype of influenza A viruses in mice. Virus Res..

[149] Patel A., Gray M., Li Y., Kobasa D., Yao X., Kobinger G.P. (2012). Co-administration of certain DNA vaccine combinations expressing different H5N1 influenza virus antigens can be beneficial or detrimental to immune protection. Vaccine.

[150] Holman D.H., Wang D., Raja N.U., Luo M., Moore K.M., Woraratanadharm J., Mytle N., Dong J.Y. (2008). Multi-antigen vaccines based on complex adenovirus vectors induce protective immune responses against H5N1 avian influenza viruses. Vaccine.

[151] Pratt W.D., Wang D., Nichols D.K., Luo M., Woraratanadharm J., Dye J.M., Holman D.H., Dong J.Y. (2010). Protection of nonhuman primates against two species of Ebola virus infection with a single complex adenovirus vector. Clin. Vaccine Immunol..

[152] Holman D.H., Wang D., Raviprakash K., Raja N.U., Luo M., Zhang J., Porter K,R., Dong J.Y. (2007). Two complex, adenovirus-based vaccines that together induce immune responses to all four dengue virus serotypes. Clin. Vaccine Immunol..

[153] Epstein S.L., Kong W.P., Misplon J.A., Lo C.Y., Tumpey T.M., Xu L., Nabel G.J. (2005). Protection against multiple influenza A subtypes by vaccination with highly conserved nucleoprotein. Vaccine.

[154] Tompkins S., Zhao Z.S., Lo C.Y., Misplon J.A., Liu T., Ye Z., Hogan R.J., Wu Z., Benton K.A., Tumpey T.M., Epstein S.L. (2007). Matrix protein 2 vaccination and protection against influenza viruses, including subtype H5N1. Emerg. Infect. Dis..

[155] Lo C.Y., Wu Z., Misplon J.A., Price G.E., Pappas C., Kong W.P., Tumpey T.M., Epstein S.L. (2008). Comparison of vaccines for induction of heterosubtypic immunity to influenza A virus: cold-adapted vaccine versus DNA prime-adenovirus boost strategies. Vaccine.

[156] Jegerlehner A., Schmitz N., Storni T., Bachmann M.F. (2004). Influenza A vaccine based on the extracellular domain of M2: Weak protection mediated via antibody-dependent NK cell activity. J. Immunol..

[157] Wei C.J., Yassine H.M., McTamney P.M., Gall J.G., Whittle J.R., Boyington J.C., Nabel G.J. (2012). Elicitation of broadly neutralizing influenza antibodies in animals with previous influenza exposure. Sci. Transl. Med..

[158] Maeda K., West K., Hayasaka D., Ennis F.A., Terajima M. (2005). Recombinant adenovirus vector vaccine induces stronger cytotoxic T-cell responses than recombinant vaccinia virus vector, plasmid DNA, or a combination of these. Viral Immunol..

[159] Lin S.C., Huang M.H., Tsou P.C., Huang L.M., Chong P., Wu S.C. (2011). Recombinant trimeric HA protein immunogenicity of H5N1 avian influenza viruses and their combined use with inactivated or adenovirus vaccines. PLoS One.

[160] Hung P.P., Chanda P.K., Natuk R.J., Mason B.B., Chengalvala M., Bhat B.M., Molnar-Kimber K.L., Dheer S.K., Morin J.E., Mizutani S. (1990). Adenovirus vaccine strains genetically engineered to express HIV-1 or HBV antigens for use as live recombinant vaccines. Nat. Immun. Cell Growth Regul..

[161] Hsu K.H., Lubeck M.D., Bhat B.M., Bhat R.A., Kostek B., Selling B.H., Mizutani S., Davis A.R., Hung P.P. (1994). Efficacy of adenovirus-vectored respiratory syncytial virus vaccines in a new ferret model. Vaccine.

[162] Alexander J., Ward S., Mendy J., Manayani D.J., Farness P., Avanzini J.B., Guenther B., Garduno F., Jow L., Snarsky V., Ishioka G., Dong X., Vang L., Newman M.J., Mayall T. (2012). Pre-clinical evaluation of a replication-competent recombinant adenovirus serotype 4 vaccine expressing influenza H5 hemagglutinin. PLoS One.

[163] National Institutes of Health http://www.clinicaltrials.gov/.

[164] Gao Y.W., Xia X.Z., Wang L.G., Liu D., Huang G. (2006). Construction and experimental immunity of recombinant replication-competent canine adenovirus type 2 expressing hemagglutinin gene of H5N1 subtype tiger influenza virus. Wei Sheng Wu Xue Bao.

[165] Pfeiffer D.U., Minh P.Q., Martin V., Epprecht M., Otte M.J. (2007). An analysis of the spatial and temporal patterns of highly pathogenic avian influenza occurrence in Vietnam using national surveillance data. Vet. J..

[166] Les S., Dung D.H. Vaccination of poultry in Vietnam against H5N1 highly pathogenic avian influenza. http://www.aitoolkit.org/site/DefaultSite/filesystem/documents/CASE%20STUDY_07-09-09%20final.pdf.

[167] Sánchez Ramos O., González Pose A., Gómez-Puerta S., Noda Gomez J., Vega Redondo A., Águila Benites J.C., Suárez Amarán L., Toledo Alonso J.R. (2011). Avian CD154 enhances humoral and cellular immune responses induced by an adenovirus vector-based vaccine in chickens. Comp. Immunol. Microbiol. Infect. Dis..

[168] Toro H., Suarez D.L., Tang D.C., van Ginkel F.W., Breedlovea C. (2011). Avian influenza mucosal vaccination in chickens with replication-defective recombinant adenovirus vaccine. Avian. Dis..

[169] Madore D.V., Meade B.D., Rubin F., Deal C., Lynn F., Meeting Contributors (2010). Utilization of serologic assays to support efficacy of vaccines in nonclinical and clinical trials: meeting at the crossroads. Vaccine.

[170] Epstein S.L., Price G.E. (2010). Cross-protective immunity to influenza A viruse. Expert Rev. Vaccines.

[171] Chen M.W., Cheng T.J., Huang Y., Jan J.T., Ma S.H., Yu A.L., Wong C.H., Ho D.D. (2008). A consensus-hemagglutinin-based DNA vaccine that protects mice against divergent H5N1 influenza viruses. Proc. Natl. Acad. Sci. USA.

[172] Laddy D.J., Yan J., Kutzler M., Kobasa D., Kobinger G.P., Khan A.S., Greenhouse J., Sardesai N.Y., Draghia-Akli R., Weiner D.B. (2008). Heterosubtypic protection against pathogenic human and avian influenza viruses via *in vivo* electroporation of synthetic consensus DNA antigens. PLoS One.

[173] Jones F.R., Gabitzsch E.S., Xu Y., Balint J.P., Borisevich V., Smith J., Smith J., Peng B.H., Walker A., Salazar M., Paessler S. (2011). Prevention of influenza virus shedding and protection from lethal H1N1 challenge using a consensus 2009 H1N1 HA and NA adenovirus vector vaccine. Vaccine.

[174] Steitz J., Barlow P.G., Hossain J., Kim E., Okada K., Kenniston T., Rea S., Donis R.O., Gambotto A. (2010). A candidate H1N1 pandemic influenza vaccine elicits protective immunity in mice. PLoS One.

[175] Catanzaro A.T., Koup R.A., Roederer M., Bailer R.T., Enama M.E., Moodie Z., Gu L., Martin J.E., Novik L., Chakrabarti B.K., Butman B.T., Gall J.G., King C.R., Andrews C.A., Sheets R., Gomez P.L., Mascola J.R., Nabel G.J., Graham B.S., Vaccine Research Center 006 Study Team (2006). Phase 1 safety and immunogenicity evaluation of a multiclade HIV-1 candidate vaccine delivered by a replication-defective recombinant adenovirus vector. J. Infect. Dis..

[176] International Federation of Pharmaceutical Manufacturers & Associations Cell culture based vaccines. http://www.ifpma.org/resources/influenza-vaccines/influenza-vaccines/cell-culture-based-vaccine.html.

[177] Novartis Vaccines and Diagnostics Inc. Novartis Holly Springs cell culture influenza vaccine manufacturing facility first to be declared pandemic ready by U.S. government. http://www.novartisvaccines.com/newsroom/media-releases/2011/Holly_Springs_.

[178] Girard M.P., Katz J.M., Pervikov Y., Hombach J., Tam J.S. (2011). Report of the 7th meeting on Evaluation of Pandemic Influenza Vaccines in Clinical Trials, World Health Organization, Geneva, 17-18, February 2011. Vaccine.

